# Cystadenocarcinoma of the parotid: case report of a BRAF inhibitor treatment

**DOI:** 10.1186/2193-1801-2-679

**Published:** 2013-12-18

**Authors:** Sabrina Boyrie, Isabelle Fauquet, Michel Rives, Caroline Genebes, Jean-Pierre Delord

**Affiliations:** Department of Radiation Oncology, Institute Claudius Regaud, Toulouse, France; Department of Radiology, Clinique Pasteur, Toulouse, France; Department of Medical Oncology, Institute Claudius Regaud, Toulouse, France

## Abstract

**Background:**

Mutations of the proto-oncogene BRAF have been described in many cancers. Mutated BRAF causes overactive downstream signaling via MEK and ERK leading to excessive cell proliferation and survival. Their identification is of real interest because specific inhibitors have been developed.

**Methods and results:**

We report the case of a patient with an aggressive cystadenocarcinoma of the parotid with synchronous metastases. Cisplatine/5FU chemotherapy associated with palliative radiation therapy was used first without any efficency nor clinical improvement. A molecular analysis revealed a BRAF mutation. A compassionate treatment with a BRAF inhibitor showed very good results from the first month. The patient reported real improvement in clinical condition and pain. From an imaging point of view, computed tomographies reported a complete response on mediastinal lymph nodes and regeneration on bone metastases.

**Conclusion:**

This first report suggests BRAF could be a potent oncogenic driver in salivary gland carcinoma. It deserves a multicenter academic prospective trial to provide proof of efficiency with BRAF inhibitors in theses tumors.

## Introduction

Malignant tumours of the parotid gland are unusual. They represent fewer than 5% of tumours in the head and neck region (Spiro 1991). Salivary gland carcinomas are a heterogenous group of tumours with great diversity in histological appearance and biological behaviour. Some tumors are less malignant and have a good prognosis while others progressing to recurrence, metastases and death of the individual. Cystadenocarcinoma is a rare malignant tumour characterized by predominantly cystic growth that often exhibits intraluminal papillary growth. These tumours, which have no identified risk factors, occur mainly in the major salivary glands and particularly in the parotid. The tumorigenesis of these tumours is still poorly understood. Despite ongoing advances in surgery, radiation therapy and chemotherapy, the 5-year survival rate for salivary gland malignancies has not changed significantly during the last few decades. But this will certainly change soon thanks to major advances in fundamental research. Since the discovery of the role of RAS oncogenes in tumorigenesis, there has been an explosion of research in the signal transduction area. A key RAS effector pathway involves the kinase cascade RAF/MEK/ERK. The B-type RAF proto-oncogene (BRAF) V600E mutation is a representative oncogenic mutation. This mutation has emerged as a prognostic variable for several tumours such as thyroid (Kim et al. 2012). Somatic mutations of BRAF have been described in Head and Neck Squamous Cell Carcinoma (HNSCC) (Weber et al. 2003). Novel agents are being designed specifically to inhibit several biochemical pathways in the pathogenesis of cancer. We report here the case of a patient with an agressive cystadenocarcinoma of the parotid with a BRAF mutation treated with a BRAF inhibitor.

## Case report

Mr B. is a 69 year-old man with no significant past medical history. He presented to our Institute with a left pre-tragus mass that appeared a few weeks ago. His general practitioner ordered a Computed Tomography (CT) scan then a Magnetic Resonance Imaging (MRI) that revealed a bifocal parotid mass. The first nodule extended into the superficial lobe of the parotid and the second through the angle of the mandible with a marked osteolysis. A nodule was also seen at the upper front of the maxillary left sinus without continuity with previous one. Clinically no mucosal lesion was identified. The fine needle aspiration biopsy of the parotid lesion exhibited glandular epithelial cells suspicious of malignancy. The biopsy then demonstrated a papillary adenocarcinoma suggesting a cystadenocarcinoma. Immunohistochemical assessment of HER2 showed grade 2+ overexpression but no HER2 gene amplification by FISH (fluorescence in-situ hybridization). Positron Emission Tomography (PET) staging showed distant metastases. Multiple bone hyperfixations were localized in C3 right costal arch, C6 with posterior wall destruction, L4 pedunculate, left femur and pelvis.

A chemotherapy was initiated on July 2011. Three cycles of cisplatin 100 mg/m^2^ and 5-fluorouracil 1000 mg/m^2^/d (J1-J4) were delivered with good tolerance. The CT scan evaluation revealed a stabilization of the mass developed at the expense of the superficial lobe of the parotid but a marked progression of bone localizations of the malar mass (33 mm versus 14 mm) and of mandible osteolysis with pathological fracture. Distant bone metastases progressed too on the MRI with an epidural extension of C6 damage and appearance of T8 and L5 lesions.

In October 2011, palliative radiation therapy was administered on the primary parotid mass, the adjacent mandible lesion and on symptomatic distant bone localizations (C6 and L4). The dose delivered was 30 Gy in 10 fractions.

The April 2012 CT scan evaluation reported a discrete regression of the irradiated parotid mass but a further progression of the malar mass measured at 53 mm. Moreover, enlarged mediastinal lymph nodes appeared in 2R, 4R and 7 as well as multiple osseous sites throughout the pelvis and spine.

Given the aggressiveness and resistance of this disease to radio-chemotherapy, an additional molecular analysis was requested to attempt to identify mutations on EGFR, KRAS and BRAF genes. No mutations were found except for BRAF. A polymerase chain reaction followed by an allele specific oligonucleotide revealed a V600E mutation of BRAF.

**Figure 1:**
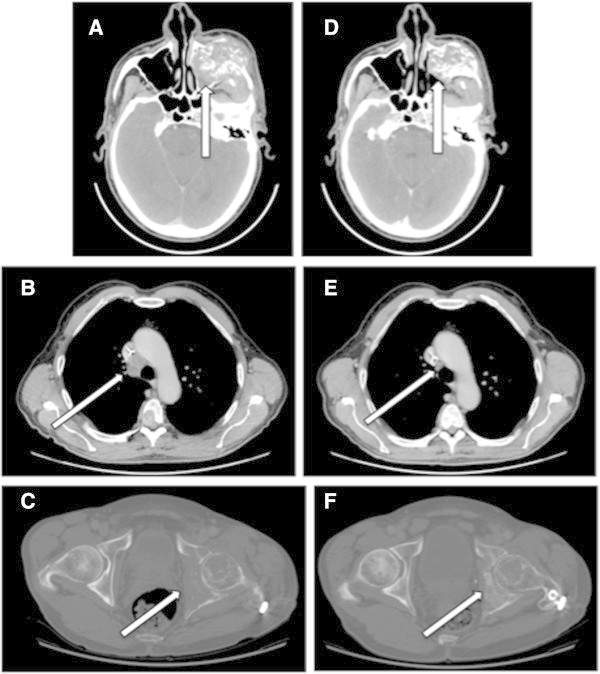
**Comparison of CT scans realized before and after two months of treatment with a BRAF inhibitor. A**, **B**, **C**: CT realized on April 2012. **D**, **E**, **F**: CT realized on June 2012 **A**, **D**: Reduction of tissue infiltration around malar bone destruction, especially on the sinusal component. **B**, **E** (after injection of contrast agent): Complete regression of mediastinal lymph nodes in station 4R. **C**, **F** (bone window): Left iliac bone regeneration. Notice cortical bone regeneration of arcuate line of ilium.

A compassionate treatment with a BRAF inhibitor (Vemurafenib) was started at a dose of 240 mg bid for 2 months after approval of internal ethic committee. The patient reported a great improvement in clinical condition from the first month. His fatigue declined allowing him to enjoy activities such as gardening. His vision improved with ptosis regression. Clinical examination found a clear regression of the malar mass. The CT scan obtained after 2 months of treatment confirmed the good clinical impression. The malar mass regressed by 42% (60 × 47 × 53 mm versus 46 × 42 × 45 mm) (Figure 1A,B). Mediastinal lymph nodes showed a complete response (Figure 1B,E). Bone regeneration was seen on pelvis (Figure 1C,F) and spine.

## Discussion

The RAF/MEK/ERK signal transduction cascade is an important mediator of a number of cellular outcomes including growth, proliferation and survival. The BRAF gene is one of the human isoforms of RAF activated by oncogenic RAS and leading to cooperative effects in cells responding to growth factor signals.

Mutations of BRAF have been described in about 15% of all human cancers (Davies et al. 2002). In contrast to melanoma or colorectal cancer (Loupakis et al. 2009), these mutations are relatively rare events in HNSCC. In their series of 89 HNSCC, Weber et al. reported only 3% with activating BRAF mutations (Weber et al. 2003). Lopez et al. showed no BRAF mutation in both sinonasal squamous cell carcinoma and intestinal-type sinonasal adenocarcinoma (Lopez et al. 2012). However BRAF mutation occurs in approximately half of papillary thyroid cancers. This mutation, correlated with high-risk clinicopathological factors and poor clinical outcome, is a poor prognostic marker in these papillary thyroid cancers (Kim et al. 2012). BRAF mutation is also a poor predictor to response in EGFR inhibitiors. Actually, potential mechanisms for the lack of response to EGFR inhibition in HNSCC include constitutive activation of signalling pathways independent of EGFR, as well as genetic aberration causing dysregulation of the cell cycle or mutation of downstream effectors such as BRAF or KRAS.

Specific inhibitors of BRAF have been developped and are now currently used in advanced melanoma with any V600 BRAF mutation. Chapman et al. reported the results of a phase 3 randomized clinical trial comparing vemurafenib with dacarbazine in 675 patients with metastatic melanoma with the BRAF V600E mutation. At 6 months, overall survival was 84% in the vemurafenib group and 64% in the dacarbazine group. Response rates were 48% for vemurafenib and 5% for dacarbazine (Chapman et al. 2011). This study provided the proof that BRAF mtations have strong oncogenic properties in this cancer. Vemurafenib is now approved for the treatment of metastatic melanomas that harbor the BRAF V600E mutation.

For salivary glands carcinoma, immunohistochemical procedures and molecular biological studies become more and more important to classify this wide variety of tumours. Overexpression of transmembrane receptors of type tyrosine kinase have been reported (HER2/neu (Cornolti et al. 2007), EGFR (Sorensen et al. 2006), C-kit in adenoid cystic carcinoma (Sorensen et al. 2006)). Target therapies against these receptors are now widely used in other cancers. Studies are ongoing in salivary gland carcinomas. BRAF mutations could also be searched in these tumours, as our case report points out. Although uncommon, it may identify a subset of patients sensitive to targeted therapy. Our case report suggests BRAF mutations are a strong oncogenic driver in salivary gland carcinoma associated with aggressiveness, chemoresistance, metastatic properties and sensitivity to BRAF inhibitors. A large collaborative academic phase II trial should be performed in order to provide proof of targeted treatment efficiency.

## Consent

Written informed consent was obtained from the patient for the publication of this report and any accompanying images.
